# Detection of differential fetal and adult expression of chloride intracellular channel 4 (CLIC4) protein by analysis of a green fluorescent protein knock-in mouse line

**DOI:** 10.1186/1471-213X-14-24

**Published:** 2014-05-28

**Authors:** VC Padmakumar, Katelyn E Masiuk, Dror Luger, Christina Lee, Vincenzo Coppola, Lino Tessarollo, Shelley B Hoover, Irina Karavanova, Andres Buonanno, R Mark Simpson, Stuart H Yuspa

**Affiliations:** 1 Laboratory of Cancer Biology and Genetics, National Cancer Institute, Bethesda, MD, USA; 2 Section on Molecular Neurobiology, Eunice Kennedy Shriver National Institute of Child Health and Human Development, Bethesda, MD, USA; 3 Mouse Cancer Genetics Program, NCI-Frederick, Frederick, MD, USA; 4 Present address: Department of Molecular Virology, Immunology and Medical Genetics, Ohio State University Medical Center, Columbus, OH, USA; 5 National Cancer Institute/NIH, Building 37, Room 4068A1, 37 Convent Drive, Bethesda, MD 20892, USA

**Keywords:** CLIC4, GFP knockin, Fetal brain, Adult brain, Kidney function

## Abstract

**Background:**

Chloride Intracellular Channel 4 (CLIC4) is one of seven members in the closely related CLIC protein family. CLIC4 is involved in multiple cellular processes including apoptosis, cellular differentiation, inflammation and endothelial tubulogenesis. Despite over a decade of research, no comprehensive in situ expression analysis of CLIC4 in a living organism has been reported. In order to fulfill this goal, we generated a knock-in mouse to express Green Fluorescent Protein (GFP) from the CLIC4 locus, thus substituting the GFP coding region for CLIC4. We used GFP protein expression to eliminate cross reaction with other CLIC family members.

**Results:**

We analyzed CLIC4 expression during embryonic development and adult organs. During mid and late gestation, CLIC4 expression is modulated particularly in fetal brain, heart, thymus, liver and kidney as well as in developing brown adipose tissue and stratifying epidermis. In the adult mouse, CLIC4 is highly expressed globally in vascular endothelial cells as well as in liver, lung alveolar septae, pancreatic acini, spermatogonia, renal proximal tubules, cardiomyocytes and thymic epithelial cells. Neural expression included axonal tracks, olfactory bulb, Purkinje cell layer and dentate gyrus. Renal CLIC4 expression was most pronounced in proximal tubules, although altered renal function was not detected in the absence of CLIC4. Myeloid cells and B cells of the spleen are rich in CLIC4 expression as are CD4 and CD8 positive T cells.

**Conclusions:**

In a comprehensive study detailing CLIC4 expression in situ in a mouse model that excludes cross reaction with other family members, we were able to document previously unreported expression for CLIC4 in developing fetus, particularly the brain. In addition, compartmentalized expression of CLIC4 in specific adult tissues and cells provides a focus to explore potential functions of this protein not addressed previously.

## Background

CLIC4 is one of the seven members in the Chloride Intracellular Channel family of proteins that include CLIC1 (NCC27), CLIC2, CLIC3, CLIC4, CLIC5A, CLIC5B, and CLIC6 (parchorin) [1-7]. CLIC4, originally named as p64H1, cloned from rat tissues, was identified as a homologue of p64 [4]. Despite its nomenclature in the Chloride Intracellular Channel group of proteins, the role of CLIC4 as a chloride channel is still under debate. While CLIC4 behaved as a channel unable to differentiate between a cation and anion in *in vitro* reconstituted membranes [8] and binds to lipid membranes and transports chloride ions [9], a physiological role for CLIC4 attributable to its chloride activity is yet to be convincingly demonstrated. Crystallographic analysis of CLIC4 structure revealed a two domain structure that has a fold very similar to Glutathione S-transferase [9].

Several cellular signals are known to induce the expression of CLIC4 including TGF-Î², LPS, p53, TNF-Î± as well as cellular stress such as DNA damage and metabolic toxicity [10-14]. CLIC4 is localized to cytoplasm, mitochondria, cell cortex, intracellular membranes and nucleus [15,16] depending on cell type studied. CLIC4 has been implicated in a variety of cellular functions including angiogenesis, apoptosis, keratinocyte and adipocyte differentiation, LPS-mediated innate immunity, macrophage deactivation and TGF-Î² signaling [10,13,17-21]. Changes in CLIC4 expression or subcellular localization are also associated with several pathological conditions including cancer, atopic dermatitis, ethylmalonic encephalopathy, alcoholism and autism, but the functional significance of these changes is still not well defined [20,22-25]. Mice lacking CLIC4 are viable and three independent groups have generated CLIC4 knockout mice and reported differing phenotypic outcomes implicating CLIC4 in endothelial tubule formation, skin and corneal wound healing and innate immunity [18,21,26].

Despite major advances in the field, a coherent narrative that ties together the numerous diverse functions attributed to CLIC4 is conspicuously absent. Previous studies analyzing protein and RNA expression from tissue lysates have revealed that CLIC4 is expressed in various tissues including kidney, liver, lungs, brain, testis and skin [27]. However the field lacks a clear in situ survey revealing cellular-level CLIC4 localization in tissue compartments as an important step for understanding its function. In part this deficiency is related to the concern that antibodies generated for CLIC4 might cross react with other closely related family members and obscure the conclusions. With this concern in mind and convinced that the CLIC4 field will benefit from a comprehensive study of CLIC4 expression during development and in various organs and tissues, we generated a knock-in mouse that expresses Green Fluorescent Protein (GFP) under the control of the native CLIC4 promoter. In this model, both low CLIC4 expression and autofluorescence interfered with direct visualization of the GFP fluorescence. By using an antibody against GFP to overcome the potential for inadvertently recognizing another CLIC family member, we were able to document differential expression of CLIC4 proteins in tissue structures and cell types during fetal development and tissue homeostasis.

## Results

### Generation of CLIC4-GFP knock-in mice

**Figure 1:**
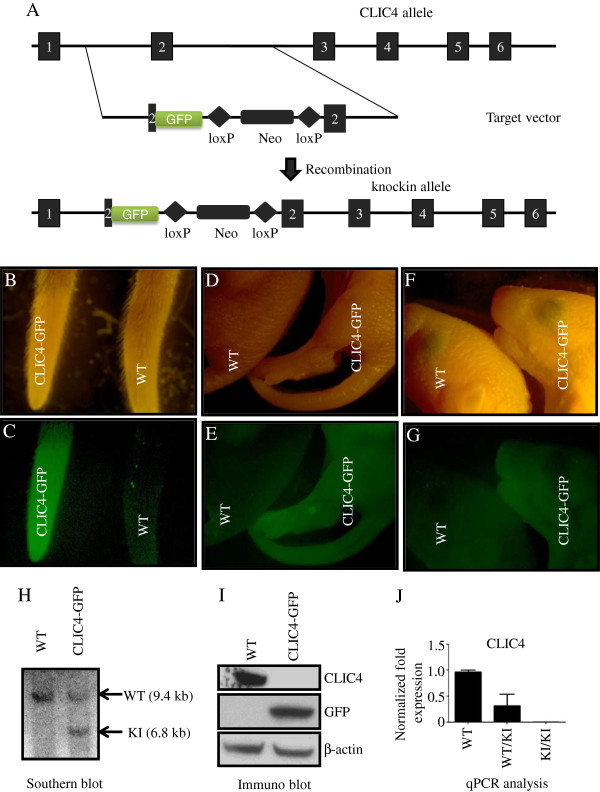
**Generation of CLIC4-GFP knock-in mouse.** Panel **A** is the schematic of the strategy used for GFP knock-in vector. **B**, **D** and **F** are light images and **C**, **E** and **G** are fluorescent images. **(B-C)**: light and fluorescent images of tails from adult WT and CLIC4-GFP mice. **(D-G)**: light and fluorescent images of WT and CLIC4-GFP new born pups. **(H)**: Southern blot from embryonic stem cells used for blastocyst injection; **(I)**: Immunoblot of protein lysates from WT and homozygous CLIC4-GFP skin using antibodies against CLIC4, GFP and Î²-actin; **(J)**: qPCR analysis of spleen lysates from WT and CLIC4-GFP heterozygous (WT/KI) or homozygous (KI/KI) mice probed for CLIC4 N-terminus.

A knock-in mouse that expresses GFP from the CLIC4 promoter was created to study the normally regulated expression of CLIC4 in mouse organs and tissues without concern for expression of other CLIC family members. The second exon was chosen for the fusion to avoid interfering with potential regulatory elements associated with the first exon (Figure 1A). The CLIC4 gene codes for a protein that consists of 253 amino acids and GFP is introduced after the 25^th^ amino acid. The GFP cassette contains Herpes Simplex Virus thymidine kinase polyadenylation signal for proper termination and processing of the recombinant transcript. This introduction of GFP in the second exon results in the termination of CLIC4 transcription from this point, thereby creating a CLIC4 knockout allele. Southern blotting confirmed positive ES cell clones that harbor the GFP in the CLIC4 locus (Figure 1H). Blastocyst injection followed by successful germ line transmission resulted in heterozygous mice carrying the GFP in one allele. Western blotting of skin lysates confirmed the expression of GFP in homozygous mice. Homozygous expression of GFP also results in the loss of expression of CLIC4 transcripts and protein (Figure 1I,J). Heterozygous mice express CLIC4 from one allele and GFP from the other. Visual examination of tails of adult mice or newborn pups under the fluorescence microscope is sufficient to identify knock-in mice (Figure 1B-G).

### CLIC4 expression in developing embryos

We evaluated the global expression patterns of CLIC4 during mid-to-late gestation development (13.5, 15.5 and 18.5 days post conception (p.c.)) using immunohistochemical analysis for GFP on midline sagittal and parasagittal sections obtained from CLIC4-GFP knock-in mice.

#### Neural tissues

**Figure 2:**
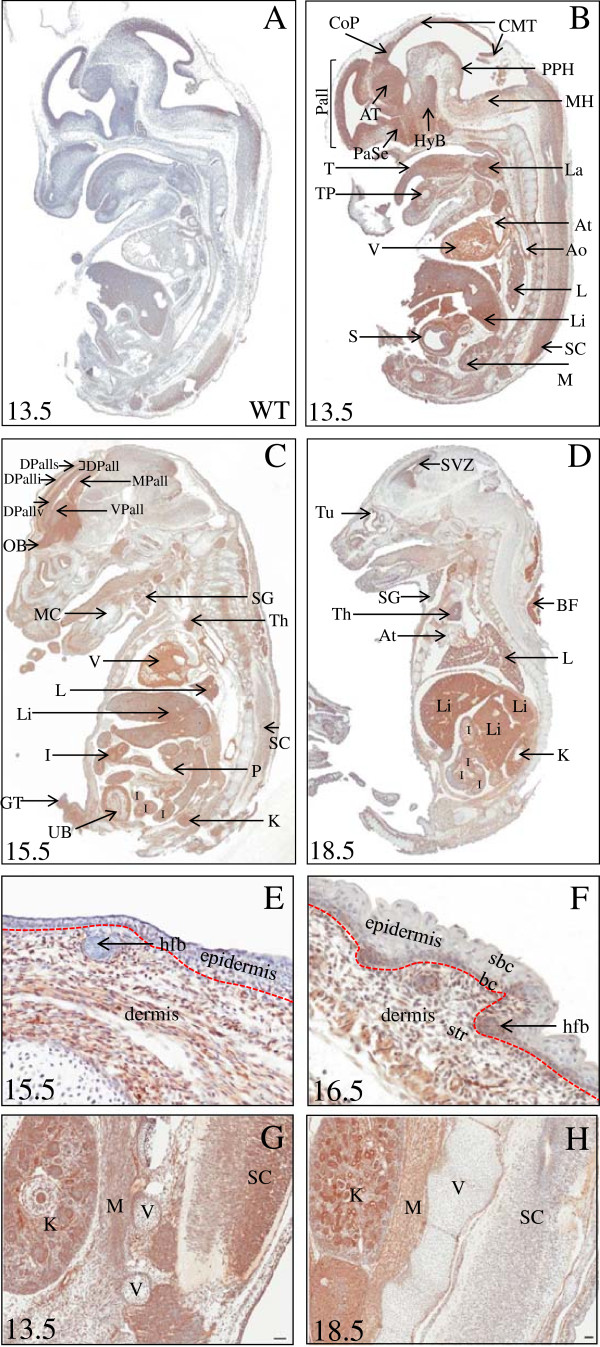
**CLIC4 expression in embryos.** 13.5. 15.5 and 18.5 days post coitus (pc) homozygous CLIC4-GFP embryos were used for the study **(A-D)**. GFP staining of 13.5 pc WT embryo is shown as a control. All embryos were stained with an antibody against GFP. CLIC4-GFP is illustrated in B-D for Pallium (Pall), alar plate of thalamus (AT), commissural pretectal region (CoP), paraseptal subpallium (PaSe), collicular midbrain tectum (CMT), basal part temporal hypothalamus (HyB), prepontine hindbrain (PPH), medullary hindbrain (MH), dorsal pallium (DPall), ventral pallium (VPall), DPall ventricular (DPallv), medial pallium (MPall), DPall intermediate stratum (DPalli), olfactory bulb (OB), regions of the central nervous system, as well as for tongue (T), tooth primordium (TP), larynx (La), cardiac ventricle (V) and atrium (At), aorta (Ao), lung (L), liver (Li), kidney (K), intestines (I), stomach (S), spinal cord (SC), cortical mesonephros (M), turbinates (Tu), Meckel’s cartilage (MC), pancreas (P), genital tubercle (GT), salivary gland (SG), thymus (Th), urachus bladder (UB) and brown fat (BF). GFP staining of integument changes at the time of stratification of the epidermis in 15.5 and 16.5 days old embryos **(E and F)** (str-stroma/dermis, bc-basal cell, sbc- suprabasal cell, hfb- hair follicle bud). CLIC4 expression as detected by anti-GFP immunohistochemistry in embryonic tissues from a E13.5 day and E18.5 day developing mouse **(G and H)**. Note that while developing kidney (K) maintains similar expression over this period of development, CLIC4 expression diminishes in myocytes (M), spinal cord (SC), and chrondrocytes vertebrae (V). Hematoxylin counterstain. Bar = 50 μm.

Temporal-regional expression of CLIC4 fluctuates in different organs, especially in the brain, where generally expression levels are high during early development and are strongly downregulated with age (Figure 2). At 13.5 p.c., (Figure 2B) analysis in the anterior to posterior axis shows that high levels of CLIC4 are expressed throughout the entire developing dorsal pallium (isocortex) that gives rise to the cortex, alar plate of the thalamus (AT), the paraseptal subpallium, basal part of the terminal hypothalamus (HyB), commissural pretectal region (CoP) and the collicular midbrain tectum (CMT); the ventral and dorsal layers of the spinal cord also express CLIC4. By contrast, structures extending from the prospective prepontine hindbrain (PPH) to the medullary hindbrain (MH) are notably devoid of CLIC4-driven GFP. By 15.5 p.c., (Figure 2C) expression throughout the diencephalon, midbrain and hindbrain becomes undetectable, and CLIC4 expression is mostly restricted to the developing ventral pallium (olfactory bulb), dorsal pallium (gives rise to frontal, occipital, insular and entorhinal cortices) and medial pallium (gives rise to hippocampus and subicullum). Interestingly, in the dorsal pallium (DPall) GFP immunoreactivity is observed in the ventricular zone (DPallv) and superficial (DPalls) strata, but not in the intermediate stratum (DPalli), suggesting that CLIC4 is expressed in the dividing neuroblast precursors in the ventricular zone and possibly during migration to the superficial layers of the isocortex. A progressive loss of GFP immunoreactivity is observed in the brain and spinal cord between 15.5 to 18.5 p.c., (Figure 2D) with the exception of very slight staining at 18.5 p.c. in the subventricular zone (SVZ), an area of limited neurogenesis in the adult brain. Taken together, our results are consistent with CLIC4 expression during neurogenesis in the developing embryo and possibly in the adult.

#### Non neural tissues

CLIC4 expression is strongly associated with development of the heart ventricles, the aorta, lung and thymus development as well as organogenesis in the abdomen, particularly intestines, pancreas, kidney and liver. There is an overall reduction CLIC4 expression as the embryo matures in the womb. The 18.5 day old embryo has lost most of the CLIC4 expression in the spinal cord and to a great extent in chondrocyte nuclei of the vertebrae compared to the 13.5 day embryo providing an example of the fact that there is diminution of expression in certain cell types in the developmental process. For comparison, the expression in the developing nephros is not as relatively different from E13.5 to E18.5 days (Figure 2G-H). CLIC4 expression in developing liver and intestinal epithelium is persistent for at least 18.5 days suggesting that CLIC4 is involved in both development and maturation of these organs. Organs in the urogenital track (with the exception of the kidney cortex) including bladder and genital ridge express CLIC4 at midgestation that diminishes near birth. Development of the tongue and salivary glands in mid-gestation is strongly associated with CLIC4 expression that wanes as the embryo matures. The development of a stratified integument at day 16.5 is associated with expression of CLIC4 only in the basal cell compartment and dermal cells with hair follicle buds expressing abundant CLIC4 on day 16.5 pc but not on day 15.5pc (Figure 2E-F). An interesting association of CLIC4 expression in conjunction with the development of brown fat (Figure 2D) starting around day 15.5 suggests a role for CLIC4 in adipogenesis.

### Expression of Clic4 transcripts in the adult brain

**Figure 3:**
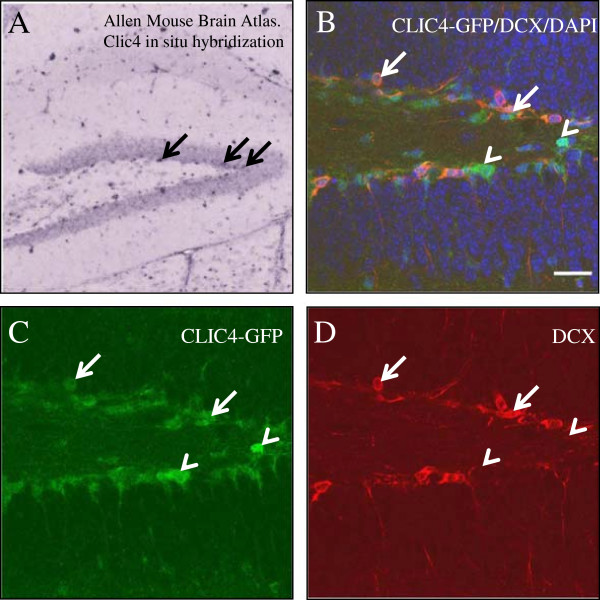
**CLIC4 is expressed in neuroprogenitor cells in the subgranular zone (SGZ) of the hippocampal dentate gyrus. (A)** Image downloaded from Allen Mouse Brain Atlas [48] showing cells expressing Clic4 transcripts in the SGZ (arrows). **(B)** Double immunofluorescence for CLIC4-GFP (green) and doublecortin (DCX, red) in the adult mouse dentate gyrus; cell nuclei are labeled by DAPI (blue). **(C, D)** Most of the SGZ cells are positive for CLIC4-GFP and, a subset of these neuroprogenitor cells (arrows) co-express DCX. Samples of GFP-positive neuroprogenitor cells not expressing DCX, which are likely to represent early progenitors, are marked with arrowheads.

To determine the pattern of CLIC4 expression in the adult brain, we used the Allen Brain Atlas (see Acknowledgements) that utilizes in situ hybridization histochemistry (ISH) with labeled cRNA probes as a guide because GFP immunostaining of adult brain tissue was difficult to detect in specific cell types. Analysis of para-sagittal sections indicates that expression of Clic4 transcripts in the adult brain is relatively low and sparse (see Additional file 1: Figure S1). Most of Clic4 expression is observed in areas rich in axonal tracks in the forebrain (i.e. corpus callosum) and cerebellum, suggesting that adult expression may be prominent in oligodendrocytes (Additional file 1: Figure S1D). The scattered expression in the neocortex and striatum in small cellular nuclei suggest expression in astrocytes. However, there are patterns of Clic4 hybridization consistent with expression in neurons. In particular, there is prominent Clic4 expression in the lateral septal nucleus (Additional file 1: Figure S1D) - an area rich in cholinergic neurons that is associated with reinforcement behaviors and reward. Other areas consistent with adult expression of Clic4 in neurons are the olfactory bulb and the cerebellum (Additional file 1: Figure S1B, C). In the olfactory bulb most of the hybridization is confined to the mitral cell layer and, to a lesser extent, the granular cell layer. In the cerebellum Clic4 expression is restricted to the Purkinje cell layer. Interestingly, Atlas data indicated that Clic4 expression in the hippocampus is observed in the dentate gyrus, where it is localized to the subgranular zone (SGZ), an area where the adult neurogenesis takes place (Figure 3). In contrast to other brain areas, we were able to obtain more defined immunostaining for GFP in the newly generated dentate neurons, which are small undifferentiated granule neurons devoid of extensive cell processes. As shown in Figure 3B, there is excellent overlap of CLIC4-GFP (Figure 3C) with doublecortin (Figure 3D), a widely used marker for newborn neurons in the adult dentate gyrus [28]. Another area known to generate adult-born neurons is the olfactory migratory stream, where Clic4 expression is also prominent (data not shown). Taken together, the prenatal and adult analysis of Clic4 expression in CLIC4-GFP mice and by ISH, respectively, suggest that CLIC4 expression is associated with early stages of neurogenesis.

### CLIC4 expression in adult organs

**Figure 4:**
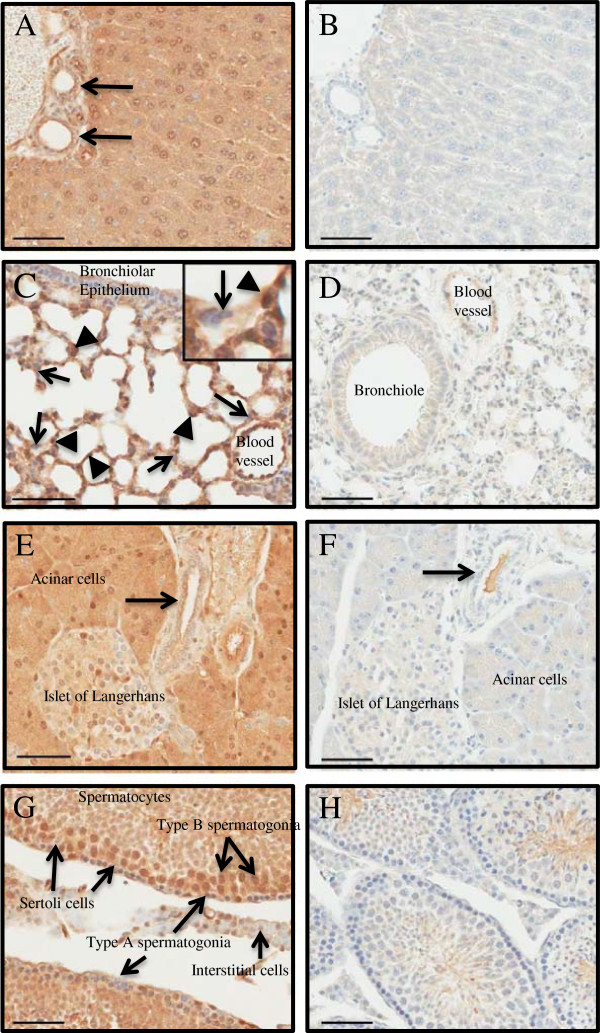
**CLIC4 expression in organs from 4 week old WT and homozygous CLIC4-GFP mice.** The left panels show GFP staining representing CLIC4 expression in liver **(A-B)**, lung **(C-D)**, pancreas **(E-F)** and testis **(G-H)**. The right panels are the corresponding organs from WT control mice treated with anti-GFP, indicating no specific GFP expression. **(A-B)**: Most liver cell types exhibit expression (see also Table 1 for additional clarification). Arrow indicates labeling of bile ductular epithelium. **(C-D)**: CLIC4 is evident in pulmonary endothelia including those lining blood vessels and within the alveolar septae (arrowheads). The alveolar pneumocytes (arrows, and see inset photomicrograph) and bronchiolar epithelia lack expression. **(E-F)**: Pancreatic islet cells are heterogeneously immuno-labeled while exocrine acinar cells are uniformly positive. Pancreatic ductular epithelia are labeled (arrow) (see also Table 1) (arrow = duct). **(G-H)**: Type A spermatogonia exhibit variable labeling at the margins of seminiferous tubules, while adluminal type B spermatogonia and spermatocytes express CLIC4, although the latter cells have variable and less pronounced signal. Sertoli cells heterogeneously express CLIC4-GFP with few cells labeling. Interstitial cells are also heterogeneously labeled, (see also Table 1). Hematoxylin counterstain. Bar = 50 um.

**Figure 5:**
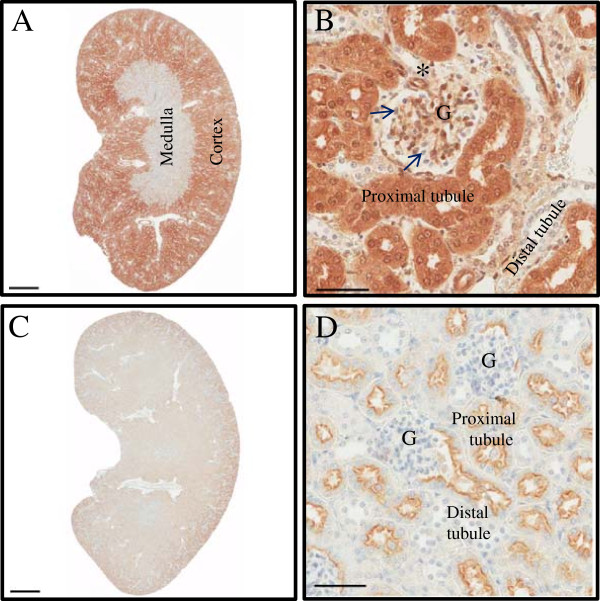
**CLIC4 expression in kidney of 4 week old mice.** GFP staining in homozygous CLIC4-GFP knock-in **(A-B)** and WT mouse **(C-D)**. Panels on the left show the entire organ, where CLIC4 expression is uniquely present in the renal cortex while absent in the medullary parenchyma. CLIC4 pattern of expression includes endothelial cells in arterioles (*) and glomerular tufts (G) (brown chromogen). Proximal tubule epithelia are intensely labeled while distal tubular epithelia are not labeled. Note glomerular visceral podocytes lack immunolabeling as well (arrows). Panel below is kidney from WT control mouse treated with anti-GFP, indicating lack of specific CLIC4-GFP expression. The signal apparent in **(D)** represents nonspecific sticking of antibody to the proximal tubule microvillus border. Hematoxylin counterstain. Bar = 50 um **(B and D)**.

We expanded our study to analyze the expression of CLIC4-GFP in adult organs (Table 1). Probing of tissue lysates had indicated that CLIC4 is expressed in many organs and tissues [27] but subcellular patterns had not been studied. Liver is one of the highest expressing organs and immunostaining confirms diffuse staining in virtually all hepatocytes and bile ductules (Figure 4A-B). Pulmonary CLIC4 is abundant in endothelia, particularly in alveolar septal endothelial cells. However, alveolar pneumocytes and bronchiolar epithelia do not express CLIC4 (Figure 4C-D). Abundant CLIC4 staining is detected in pancreatic acinar cells but cells in ductules and Islets of Langerhans are heterogeneously labeled (Figure 4E-F). CLIC4 is highly expressed in testes where expression is higher in type B spermatogonia and spermatocytes (Figure 4G-H). Sertoli and interstitial cells are heterogeneously labeled for CLIC4. As suggested by the findings in mouse embryos, high levels of CLIC4 are detected in renal cortex and glomerular tuft endothelial cells with a noticeable absence in the medullary tubular epithelia (Figure 5A). Within the cortex, CLIC4 is detected in the proximal tubules and largely absent from distal tubules suggesting a function in reabsorption (Figure 5B). A comprehensive analysis of CLIC4 expression using both homozygous and heterozygous mice was performed and the results are listed in Table 1. In summary, CLIC4 is expressed in a wide variety of cell types in major organs and in endothelial cells of many organs. Of interest also is the segmental expression of CLIC4 in lateral and ventral prostate but lack of detectable expression in anterior and dorsal prostate. The absence of CLIC4 in adult skeletal muscle, urinary bladder, ureter, and tissues of the digestive system among others is notable. Tissues were not available for ovary and uterus. Encouraged by the interesting pattern of expression of CLIC4 in kidney, we asked if there is dysfunction in the kidney in mice where CLIC4 has been genetically deleted [26]. Analysis of 22 months old female mice (3 mice per group) revealed a modestly reduced body weight in CLIC4 KO mice but the difference did not achieve significance. We also did not observe any difference in relative weight of the kidney between the genotypes. Kidney function tests performed on serum from the same group of mice showed a trend for elevation of BUN in knockout mice but this did not achieve statistical significance (Table 2).

**Table 1:** CLIC4 expression in adult tissue structures and cell types

**CLIC4-GFP-KI mouse tissue**	**Major cell type or structure labeled**	**CLIC4-GFP labeling homozygote**	**CLIC4-GFP labeling heterozygote**
Liver	Hepatocytes	+	0/+
	Sinusoidal lining cells	+	0/+
	Bile ductules	+	0/+
	Arteriolar smooth muscle*	+	+
	Venules*	+	+
Lung	Alveolar septae endothelium	+	+
	Alveolar pneumocytes	0	0
	Venular endothelium	+	+
	Bronchiolar arteriolar smooth muscle*	0	0
	Pulmonary arteriolar and venular smooth muscle	+	+
	Bronchiolar epithelium	0	0
	Bronchiolar smooth muscle	0/+	0/+
Kidney	Glomerular tufts- endothelial cells	+	+
	Glomerular visceral podocytes	0	0
	Proximal convoluted tubules	+	0/+
	Distal convoluted tubules	0	0
	Medullary endothelium	+	0/+
	Medullary collecting ducts	0	0
Spleen	Red pulp tissue - hematopoietic cells	+	0
	White pulp -Individual cells, nonlymphoid monocytoid †	+	0
Heart	Cardiomyocytes	Diffuse labeling +	0/+
	Coronary and pulmonary artery and vein smooth muscle	+	0/+
Thymus	Thymic medullary epithelial cells	+	+
	Medullary thymocytes	0/+	0/+
Trachea	Tracheal mucosa	0	0
	Perichondrium	0/+	0
	Chondrocytes	+	0/+
Pancreas	Islet cells	Heterogeneous: +	0
	Exocrine	+	0/+
	Ductules	+	0/+
Stomach	Parietal cells	na	0
	Chief cells	na	0
	Smooth muscle	na	0/+
Esophagus		0	0
Jejunum	Villus epithelium	na	0
	Lamina proprial endothelium	na	0/+
	Smooth muscle	na	0/+
Skeletal Muscle	Myocytes	0	0
Urinary Bladder	Mucosal transitional epithelium	0	0
	Smooth muscle	0	0
Ureter	Mucosal transitional epithelium	0	0
	Smooth muscle	0	0
Urethra	Mucosal transitional epithelium	Variable labeling +	0
Testis	Spermatagonia type A	Variable labeling +	na
	Spermatogonia type B	+	na
	Sertoli cells	0/+	na
	Interstitial cells of Leydig	0/+	na
	Vas deferens	0	0
Accessory Sex Glands	Anterior prostate	0	0
	Dorsal prostate	0	0
	Lateral and ventral prostate	+	0/+
	Coagulating gland	+	0/+
	Bulbourethral gland	+	na
Adrenal Gland**		na	+
Thyroid Gland	Follicular epithelium	na	Variable labeling 0/+

The table lists major cell types and structures labeled by anti-GFP staining in both homozygous and heterozygous mice. SCORING: 0 = Negative, + = Cell type/tissue positive, 0/+ = Heterogeneous immunolabeling (only some cells label) in homozygous; weak immunolabeling in heterozygous. NOTES: *Vascular smooth muscle myocytes and endothelium were uniformly positive in all tissues unless otherwise noted (homozygous). na = Tissue not available. † Cells morphologically appear to be dendritic cells. **Outer zone of adrenal medullary pheochromocytes subjacent to the corticomedullary junction. Cell types not listed within a given tissue were negative for expression except for vascular endothelium which was labeled throughout all tissues in the homozygous but not universally so in the heterozygous.

**Table 2:** Kidney functions tests of WT and CLIC4 KO mice

	**WT**	**CLIC4 KO**	**P value**
Weight	33 g	29.3 g	.0578
Relative kidney weight	0.61%	0.70%	.066
Glucose	161 mg/dL	131 mg/dL	.4263
Potassium	5.6 mmol/L	4.4 mmol/L	.3382
Sodium	152 mmol/L	153 mmol/L	.4766
Chloride	116 mmol/L	114 mmol/L	.2746
BUN	21 mg/dL	27 mg/dL	.0872
Albumin	2.7 g/dL	2.5 g/dL	
Calcium	2.24 mmol/L	2.34 mmol/L	.3935
Magnesium	1.28 mmol/l	1.18 mmol/l	.4961
Protein	5 g/dL	4.8 g/dL	.5462
Uric Acid	1.5 mg/dL	1.06 mg/dL	.3540

The table shows mean total body weight, kidney weight, kidney weight as a % of body weight and serum analysis for kidney function in age and sex matched 3 WT and 3 CLIC4 KO mice. The mice were 22 months old. Student *T* test was used to evaluate statistical significance. (The absence of p value in albumin is due to the absence of a value for one of the three samples for that parameter).

### CLIC4 expression in hematopoietic tissues

**Figure 6:**
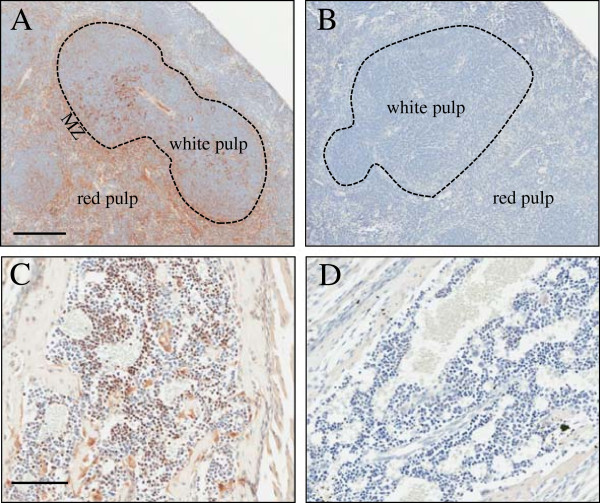
**CLIC4 staining in hematopoietic tissues of 4 week old mice.** Panel **A** and **B** show staining with GFP antibody in homozygous CLIC4-GFP and WT spleen respectively. MZ stands for marginal zone rich in macrophages. Panels **C** and **D** show GFP staining of embryonic marrow from CLIC4-GFP and WT mice. CLIC4-GFP expressing hematopoietic precursor cells are evident in panel **C**. In addition, immunolabeled osteoblasts and osteoclasts were present around foci of endochondral ossification in E19.5 day embryo vertebrae (not shown). Scale bar for panel **A** and **B** correspond to 200 μm and for panels **C** and **D** to 100 μm.

**Figure 7:**
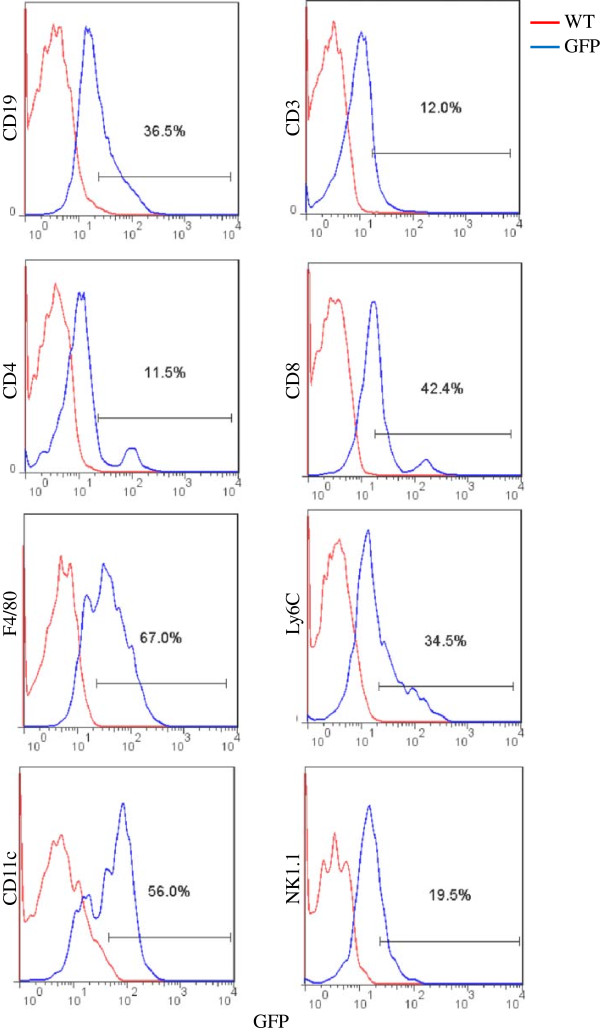
**CLIC4 expression in spleen derived hematopoietic cells from 4 week old mice.** WT and homozygous CLIC4-GFP spleen cells positive for specific markers were compared together in a histogram. The simultaneous comparison is performed to exclude potentially false positive green autoflourescence. Rightward shift of the cells from CLIC4-GFP spleen indicate GFP fluorescence. The horizontal gate on each panel shows the percentage of cells with GFP fluorescence obtained from respective positive cells gated in Additional file 2: Figure S2. The data reveal expression of CLIC4 in B cells (CD19), T cells (CD3), Helper T cells (CD4)*, cytotoxic T cells (CD8)*, NK cells (NK1.1), dendritic cells (CD11C), macrophages (F4/80) and monocytes (Ly6C). *presented as percentage of CD3 T cells.

We and others have noted the high expression of CLIC4 in macrophages prompting us to expand our analysis to explore the expression of CLIC4 in hematopoietic tissues. Immunohistochemistry of embryonic bone marrow and adult spleen confirmed high CLIC4 expression (Figure 6A-D). In the spleen, CLIC4 was most abundant in macrophages present in the marginal zone between the red and white pulp. To distinguish the cell types expressing CLIC4 in the spleen in more detail, we exploited the high sensitivity of flow cytometry to study hematopoietic subpopulations that could be distinguished directly by GFP fluorescence. Analysis of CLIC4 expression in the different splenocyte subpopulations was measured by staining with specific markers. Cells isolated from WT and CLIC4-GFP spleens were first gated using specific markers for hematopoietic cells (Additional file 2: Figure S2). These positive cells from WT and CLIC4-GFP spleen were simultaneously compared for GFP fluorescence (Figure 7). High expression of CLIC4 was found in cells from myeloid origin (macrophages (F4/80), monocytes (Ly6C) and dendritic cells (CD11c)), and in B cells (CD19). T cells (CD3), in general, showed lower GFP expression. Further analysis of the two major T cell subpopulations showed differential expression of CLIC4 (lower in CD4 and higher in CD8).

## Discussion

CLIC family members are highly conserved throughout both vertebrate and invertebrate evolution [29] and have been implicated in multiple cellular functions. CLIC proteins are amphi-morphic, existing in both soluble and membrane associated conformations at least in part determined by the redox state of the intracellular milieu. This duality of structure has made it difficult to assign a single function that encompasses all the experimental observations involving CLIC proteins. The most experimentally supported direct result of a CLIC protein action is in the formation of the gut lumen in *C. elegans* (the CLIC homologue exc-4 gene product) and tube formation in mouse blood vessels (CLIC4); while the loss of exc-4 in C. elegans causes expansion of the gut lumen into a large cyst, deficiency of CLIC4 in mice and vascular endothelial cells prevents normal vascular tubulogenesis [17,18,30,31] Mice devoid of CLIC4 also have skin and cornea wound healing defects. It has been suggested that the vascular abnormalities result from a defect in acidification of vacuoles possibly dependent on ion transport functions of CLIC4 while the wound healing defects likely result from the involvement of soluble CLIC4 in TGF-Î² signaling [18,26]. Although prior tissue lysate analyses have revealed presence of CLIC4 protein or transcripts in skin, lung, liver, kidney, heart, brain, spleen, testis, an extensive immunohistochemical analysis for cellular or developmental expression has been lacking [26,27]. We have designed a simple but convincing genetic tool to study the expression of mouse CLIC4 protein in situ in the absence of concern for cross reactivity with other closely related family members. This has provided the first look at CLIC4 expression during mammalian fetal development and a chance to probe for CLIC4 expression in a variety of adult organs, tissues and cells.

A striking example of differential expression of CLIC4 is in the brain, as determined using the CLIC4-GFP knock-in mice (Figures 2, 3 and Additional file 1: Figure S1). Interestingly, the original isolation of CLIC4 protein (called p64H1 at the time) was from rat brain [32]. Subsequently, CLIC4 was associated with dense core vesicles in neurons from the rat hippocampus [33] and detected as a binding partner for dynamin in rat brain lysates [34]. As we show, CLIC4 is abundant in developing brain in mid-gestation but is strongly downregulated during later fetal development. CLIC4 continues to be expressed in adult brain in areas associated with adult neurogenesis (Figure 3), such as the subgranular layer of the dentate gyrus [28] and the rostral olfactory migratory stream [35]. These patterns of expression suggest a potential role of CLIC4 in regulating early stages of neurogenesis.

Members of the CLIC gene family are important modulators of ethanol-mediated behaviors from invertebrates to mammals and, therefore, could constitute novel targets for treating alcohol abuse. A recent genetic linkage and association study in human and mice identified CLIC4 as the gene most highly associated with behavioral responses to alcohol [24,36]. In this regard the prominent expression of CLIC4 observed in the lateral septum is important, because this brain structure is strongly responsive to acute alcohol treatment, desensitizes with subchronic alcohol exposure (4 days) and is reactivated during withdrawal [37], suggesting a potential role in the addiction process. Future experiments using CLIC4 KO mice could help elucidate its potential role in alcohol addiction. CLIC4 may participate in other forms of brain pathology, as selective CLIC4 upregulation is detected in brain after experimentally induced stroke [38] and chromosomal transposition of the CLIC4 gene was detected in a patient with autism [25]. Together, these finding suggest that deeper analysis of CLIC4 in neuronal function and pathology will yield new insights into those processes.

While we did not probe for CLIC4 in fetal or adult eye, CLIC4 is known to be enriched in the apical microvilli of retinal pigment epithelial cells and necessary for their maintenance. Targeted deletion of CLIC4 from retinal pigment epithelial cells reduces adhesion of these cells with photoreceptor outer segments [39]. Clic4 transcripts are also elevated in mouse retinas after intense light damage [40]. The development of brown fat is a relatively late occurrence in fetal mice and associated with strong expression of CLIC4 protein (Figure 2D). This is consistent with an earlier report that Clic4 transcripts increase rapidly in 3T3L1 cells induced to differentiate into adipocytes in vitro [41]. Previous studies had also identified the presence of CLIC4 along with CLIC1 and CLIC5 in bovine spermatozoa [42]. Our probing in mouse testes indicates a predominant expression of CLIC4 in spermatogonia implying an early role for the protein in spermatogenesis. These revelations indicate that further analysis of the function of CLIC4 in both adipogenesis and spermatogenesis is warranted.

Among the more interesting distributions of CLIC4 in adult tissues are in the kidney and hematopoietic system. Human CLIC4 was first cloned from a human pancreatic cell line and derived antibody indicated strong staining in the human kidney with selectivity for the proximal tubules [43]. Our results confirm that localization and indicate the participation of CLIC4 in renal embryogenesis and morphogenesis. Furthermore, the strong compartmentalization of CLIC4 protein in proximal tubules suggests an important role in renal function. Confirming the significance of CLIC4 in renal function, it was recently shown that the ablation of CLIC4 predisposes mice to acute renal injury induced by folic acid without affecting recovery or fibrosis. In that study, CLIC4 KO mice exhibited renal dysfunction with small kidneys (males only), proteinuria and reduced glomerular counts at baseline [44]. The CLIC4 mice in our study did not have obvious renal dysfunction (Table 2) suggesting that mouse genetic background or age may have a significant modifying effect on the consequences of CLIC4 loss. Considering these findings, further analysis of CLIC4 function in the kidney and possible dysfunction in human kidney diseases needs to be addressed. Since both CLIC1 and CLIC5 have also been detected in kidney lysates but unchanged in the absence of CLIC4, the overlapping roles of CLIC family proteins in general are likely to contribute to renal homeostasis and disease [45].

Recent interest in CLIC4 in innate immunity comes from the high expression detected in macrophages [13,21]. Our mouse model confirms this observation revealing high expression in the different innate immune cells in the spleen from myeloid origin. The differential CLIC4 expression between innate immune cells and lymphocytes, between B cells and T cells and between CD4 and CD8 T cells expand the potential of this protein as an immune modulator. The development of spontaneous skin erosions in aging CLIC4 KO mice [26] suggests a potential function for the protein in autoimmunity. This possibility is currently being explored in our laboratory. In fact, CLIC4 expression is suppressed in effector T cells when they encounter Treg cells in vitro [46].

Despite the ubiquitous expression of CLIC4 in fetal development of essential organs and vital adult tissues, CLIC4 knockout mice develop normally, are fertile and present only limited phenotypes. This is most likely due to the fact that one or more CLICs under homeostatic conditions compensate for the loss of CLIC4 in different organs. It is also likely that there are tissues and organs where compensation is incomplete and inefficient. It is possible that deficiencies in CLIC4 function will be revealed when these cells, tissues or organs are challenged with appropriate experimental conditions. Several such findings have already been reported including a function for CLIC4 in endothelial tubulogenesis, wound healing and innate immunity [18,21,26]. We believe that our study will encourage investigation of the functions of CLIC4 in hitherto unexplored organs in which high levels of CLIC4 expression are observed and compartmentalized. The CLIC4-GFP mouse will be a valuable tool for researchers interested to study the expression of CLIC4 in less abundant cell types that are difficult to study using standard detection techniques but could be sorted by GFP based flow cytometry.

## Conclusions

The ability to examine unambiguous differential expression of CLIC4 in vivo using a unique genetically modified mouse model has revealed previously unknown patterns of CLIC4 expression in fetal development and adult tissues. In particular CLIC4 appears to participate in mid gestational brain differentiation, the most deterministic period of specialization, and in adult brain neurogenesis. These revelations indicate that further examination of CLIC4 function in neuronal tissue could implicate dysfunctional CLIC4 regulation in brain pathology. Adult organs where novel CLIC4 patterns have been revealed also suggest further functional studies are indicated. For example, differential expression of CLIC4 in spermatogonia as opposed to developing spermatocytes suggests a role in testicular function, restricted staining in renal proximal tubules suggests a role in reabsorption, and widespread expression in pancreatic cells suggest an exocrine related function. Prior studies have indicated that CLIC4 is important in acute immune responses, but we now show that CLIC4 is expressed in the lymphocytic lineage indicating a potential function in adaptive immunity. Together this survey has pointed to new directions of study for further analysis of this member of a highly conserved protein family in normal physiology and disease pathogenesis.

## Methods

### Animal studies

Mouse studies were performed under a protocol approved by the National Cancer Institute (NCI) and NIH Animal Care and Use Committee.

### Insertion of GFP into the CLIC4 locus

p-Bluescript-light vector containing the second exon of the CLIC4 locus including the surrounding homology arms which was originally used for the generation of CLIC4 KO mice was used for in frame fusion of GFP [26,47]. An Emerald Green Fluorescent Protein (EmGFP) coding sequence along with a stop codon followed by the Thymidine Kinase polyadenylation signal was amplified from pcDNA™6.2/C-EmGFP TOPO (Life Technologies, Carlsbad, CA, USA) and recombineered into the second exon following the codon for amino acid 25 of the CLIC4 gene. A neomycin cassette flanked by loxP sites was engineered subsequent to the GFP cassette. The targeting vector (BSP080) was linearized by NotI digestion and electroporated into mouse embryonic stem (ES) cells v6.4 (hybrid 129Sv/C57BL/6). ES cell clones were subsequently selected with G418 and gancyclovir. Southern blotting using probes that bind to regions external to the 5’ and 3’ homology arms identified positive clones. Chimeric mice were generated by injecting positive clones harboring the GFP knock-in. Germ line transmission was achieved from mating chimeric mice to C57BL/6 wild type (WT) females. PCR is routinely used to genotype the mice. The oligos used for the genotypings are, TAGAGAGGCACAGGAAAGCCCATT (OSP208), CAGTTTCCAATGCTTTCACCATC (OSP209) and AACTTCAGGGTCAGCTTGC CGTA (OSP210). WT and knock-in mice produce 180 bp and 300 bp PCR products respectively.

### Southern blotting

Genomic DNA from ES cells was digested with PstI and the digest was run on an agarose gel. The gel was rinsed in water and denatured in a solution containing 1.5 M NaCl and 0.5 M NaOH for 30 minutes. After rinsing in water, the gel was neutralized for a period of 30 and 15 minutes in a solution containing 1M Tris, 3M NaCl. After rinsing, gel was equilibrated in transfer buffer (10X Saline-Sodium Citrate (SSC) buffer). The digested DNA was then transferred to Hybon XL nylon membrane (#RPN119B, GE Healthcare Life Sciences, Uppsala, Sweden) in transfer buffer. After overnight transfer, the membrane was cross-linked in UV Stratlinker 2400 at 254 nm. The membrane was hybridized using a 5’ probe. The hybridization mix was prepared by adding the probe, dATP, dTTP, dGTP and (Î±32-p) dCTP and klenow fragment using the random primers DNA labeling system (#18187-013, Life Technologies, USA). The probe was added to the membrane in Rapid Hyb buffer (#RPN1635, GE Health Care Life Sciences), which was pre-hybridized at 65°C with the same buffer for one hour. The hybridization was performed overnight at 65°C. The membranes were washed with a solution containing 0.1% SSC and 0.1% SDS and bands visualized by autoradiography. The expected band size for WT was 9.4 kb and knock-in was 6.8 kb. The 5’probe was amplified from region upstream of the second exon of CLIC4 gene using the oligos OSP175 and OSP176. The sequences of the oligos are as follows; OSP175 : AGGGTGGGCATCAGTGCTGTTAATAGCAG and OSP176: TGCTTAGACAGAACATT AG TCAGGAAAA C.

### qPCR

RNA from spleen cells was isolated using Trizol reagent according to Manufacturer’s instructions (#15596-026, Life Technologies). Genomic DNA contamination was removed by on-column digestion using columns from RNeasy mini kit (#74106, Qiagen) and RNase-free DNase set (#79254, Qiagen, Netherlands). cDNA was synthesized using SuperScript® III First-Strand Synthesis SuperMix for qRT-PCR (#11752-050, Life Technologies). Sequences of the oligos used were TGGTGAAAGCATTGGAAACT (OSP228) and GGCACAAGACTTCTTCGAGA(OSP229). These oligos were designed for the second and third exons of CLIC4.

### Immunoblotting

Protein lysate was isolated from skin and spleen cells. The method for isolating protein and probing for the expression of CLIC4 are described elsewhere [26].

### Immunofluorescence

Mice were transcardially perfused with 4% PFA in 0.1 M PBS, pH 7.4. Brains were postfixed overnight in the same fixative and 50 μm sections were cut on a vibratome and saved for up to 3 weeks in 0.1 M PBS with sodium azide at +4°C. Sections were blocked in 10% normal donkey serum (DGS), 0.25% Triton X-100 in 0.1 M PBS for 1 h at room temperature (RT) and incubated with primary antibodies against GFP (rat monoclonal used at 1:1000, Nacalai Tesque, Japan) and/or doublecortin (DCX) (goat polyclonal at 1:1000, SantaCruz, CA) in the blocking buffer for 24 h at +4°C with gentle rocking. Slices were washed in 0.1M PBS with 0.25% Triton X-100 for at least 30 min before incubation with Alexa-488 and Cy3-conjugated donkey secondary antibodies (Jackson ImmunoResearch Laboratories, PA) for 90 min at RT in blocking buffer. After extensive washes in PBS with 0.25% Triton X-100, sections were mounted on gelatin-coated slides, dried, and mounted in Mowiol-DABCO. Fluorescence was analyzed on a Zeiss LSM 510 Meta confocal microscope (Zeiss Microimaging) at 20X and 40X magnifications. Images were adjusted for overall brightness and contrast in Adobe Photoshop (Adobe Systems).

### In situ hybridization

In situ data was used from publicly available Allen Atlas (http://mouse.brain-map.org/)[48]. We analyzed consecutive sagittal and coronal sections of adult male C57Bl/6J mouse brains (P56) hybridized with a 991 nucleotide cRNA probe corresponding to CLIC4 (NCBI Accession # NM_013885.2; bp: 1228-22219); the two experiments were performed independently as indicated by the documentation. Hybridization signals were observed to correspond to the same anatomical structures in both the sagittal and coronal sections. The specificity and relative strength of signals were confirmed using the expression mask function.

### Immunohistochemistry

Embryos and organs were fixed in 10% NBF (neutral buffered formalin) overnight and paraffin sections were made by Histoserv, Inc, Germantown, MD. Sagittal sections on three different planes for each stage of embryo starting from 13.5 days to 18.5 days were used for the staining. GFP staining was performed by the National Cancer Institute Pathology/Histotechnology Laboratory. 1;1000 dilution of anti-GFP antibody (ab6556, Abcam, Cambridge, England) was used for the staining. Immunohistochemical signal was developed in tissues using 3,3'-diaminobenzidine (DAB, brown) chromogen.

### Preparation of single cell suspensions for flow cytometry

Single cell suspensions for flow cytometry and FACS were prepared in complete RPMI 1640 medium containing 10% heat-inactivated Fetal Bovine Serum (FBS) (GIBCO), 50 μM 2-mercaptoethanol and penicillin/streptomycin (GIBCO). Spleens were then mashed with the plunger of a 1 ml syringe through a 70 μm cell strainer (BD Falcon). Single cell suspensions were washed with ice cold PBS, and remaining red blood cells were lysed in ACK Lysis Buffer. Cells were recovered in complete medium, filtered through 40 μm cell strainers, counted with a Cellometer (Nexcelom Bioscience, MA) and maintained on ice for further analysis. ACK (Ammonium-Chloride-Potassium) lysis buffer was prepared by adding 8.3 g of NH_4_Cl, 1 g of KHCO_3_, 200 μL of 0.5M EDTA in 1 liter of water and pH adjusted to 7.2 - 7.4.

### Flow cytometry and FACS analysis

Cells surface Fc receptors were blocked by incubation with anti-Fc (CD32/CD16, clone 93) (#422301, BioLegend, CA) for 15min. Mouse cells were stained with the relevant antibodies in FACS buffer (PBS with 10% FBS and 0.5 mM EDTA) for 30 min at 4°C, washed and stained with 7-AAD (7-Aminoactinomycin D) (#420403, Biolegend) to enable exclusion of dead cells. Anti-mouse antibodies used for FACS staining (Biolegend, CA) were the following: PE-CD19 (6D5), APC-Ly6C (HK1.4), APC-F4/80 (BM8), APC-CD3 (145-2C11), APC-CD4 (GK1.5), PE-CD8a (53-6.7), APC-CD11c (N418), APC-NK1.1 (PK136).

Flow cytometry was performed on a FACSCalibur (BD Biosciences, CA) instrument and analyzed with FlowJo software (FlowJo, OR). FACS analysis was done by gating on leukocytes using forward scatter and side scatter, followed by gating on live cells (negative for 7-AAD). Analysis of GFP expression in each specific immune cell population was done by gating on the positive cell marker located within the statistical bar area in histogram graph (Additional file 2: Figure S2).

### Kidney function tests

Serum from three independent aged matched littermate mice for each group was used for the analysis of kidney function tests. The tests were performed at the Department of Laboratory Medicine at the NIH with an automated hematology analyzer (CELL-DYN® 3700, Abbott).

## Competing interests

The authors declare that they have no competing interests.

## Author’s contributions

VCPK designed and created the mouse model, conceived and conducted the experiments, wrote the manuscript, KM performed immunohistochemical staining and organized the pictures, DL helped with the cell sorting and analysis, CL carried out the genotypings, VC and LT helped with the generation of the mouse model, SH and MS helped with histopathology, IK and AB carried out brain staining and SHY participated in its design and coordination and helped to draft the manuscript. All authors read and approved the final manuscript.

## Supplementary Material

Additional file 1: Figure S1 Expression analysis of CLIC4 transcripts in the adult brain using ISH. (A) Bright field image of a sagittal section of a 54d old mouse (image downloaded from the Allen Mouse Brain Atlas); boxes indicate regions of interest that are enlarged for panels B-D. (B) Clic4 expression in the olfactory bulb (OB) shown in bright field (left) and with an expression mask (right) to indicate the intensity of Clic4 hybridization (the scale from red-to-blue corresponds to high-to-low signal intensity, respectively). The highest Clic4 expression in the OB (arrows) correspond to glomerular (Gl), mitral (Mi) and granular (Gr) cell layers. (C) In the cerebellum, Clic4 expression is restricted to the Purkinje cell layer (Pu). (D) Clic4 is highly and widely expressed in lateral septal (LS) nuclei and the corpus callosum (cc), and it is detected at low levels in the hippocampus (Hi) where expression is confined to the dentate gyrus (DG). Click here for file

Additional file 2: Figure S2 Flow cytometry analysis of splenocytes from homozygous CLIC4-GFP knockin mice. Splenocytes from WT and CLIC4-GFP mice were stained with antibodies against CD19, CD3, CD4, CD8, F4/80, Ly6C, CD11c and NK1.1. The horizontal bar in each panel represents the percentage of positive cells. Positive cells from WT and CLIC4-GFP spleen are subsequently plotted in a histogram in Figure 7. Click here for file
