# Supplementary figures and images for: Detection of differential fetal and adult expression of chloride intracellular channel 4 (CLIC4) protein by analysis of a green fluorescent protein knock-in mouse line
Source: BMC Dev Biol. 2014 May 28;14:24. doi: 10.1186/1471-213X-14-24 (PMC4073518; doi:10.1186/1471-213X-14-24)

Supplementary figure 1

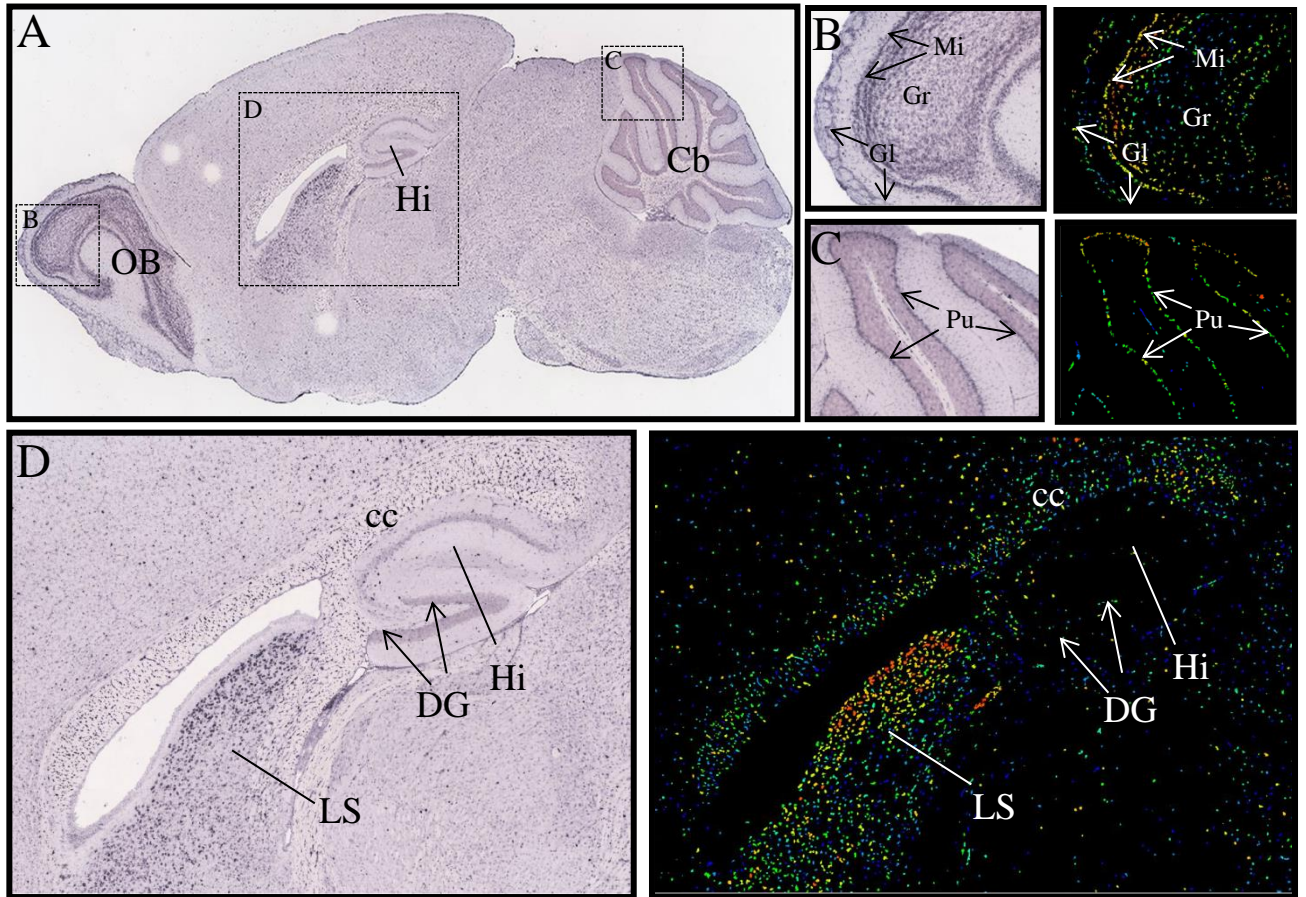

Supplement: Additional file 1: Figure S1 — Expression analysis of CLIC4 transcripts in the adult brain using ISH. (A) Bright field image of a sagittal section of a 54d old mouse (image downloaded from the Allen Mouse Brain Atlas); boxes indicate regions of interest that are enlarged for panels B-D. (B) Clic4 expression in the olfactory bulb (OB) shown in bright field (left) and with an expression mask (right) to indicate the intensity of Clic4 hybridization (the scale from red-to-blue corresponds to high-to-low signal intensity, respectively). The highest Clic4 expression in the OB (arrows) correspond to glomerular (Gl), mitral (Mi) and granular (Gr) cell layers. (C) In the cerebellum, Clic4 expression is restricted to the Purkinje cell layer (Pu). (D) Clic4 is highly and widely expressed in lateral septal (LS) nuclei and the corpus callosum (cc), and it is detected at low levels in the hippocampus (Hi) where expression is confined to the dentate gyrus (DG). [file 1471-213X-14-24-S1.pdf]

# Supplementary figure 2

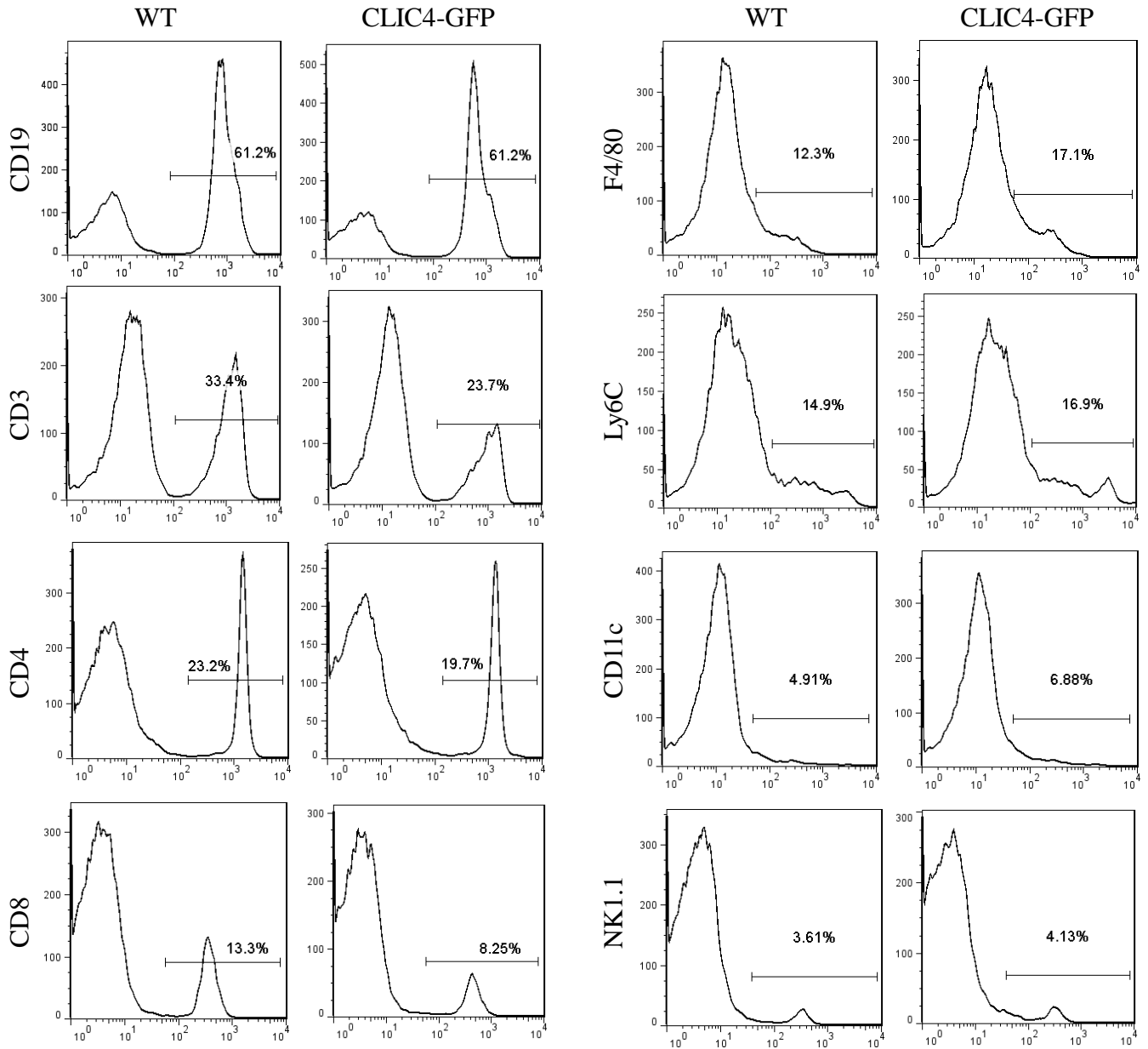

Supplement: Additional file 2: Figure S2 — Flow cytometry analysis of splenocytes from homozygous CLIC4-GFP knockin mice. Splenocytes from WT and CLIC4-GFP mice were stained with antibodies against CD19, CD3, CD4, CD8, F4/80, Ly6C, CD11c and NK1.1. The horizontal bar in each panel represents the percentage of positive cells. Positive cells from WT and CLIC4-GFP spleen are subsequently plotted in a histogram in Figure 7. [file 1471-213X-14-24-S2.pdf]
